# Effects of Noise Bandwidth and Amplitude Modulation on Masking in Frog Auditory Midbrain Neurons

**DOI:** 10.1371/journal.pone.0031589

**Published:** 2012-02-10

**Authors:** Jozien B. M. Goense, Albert S. Feng

**Affiliations:** 1 Center for Biophysics and Computational Biology, University of Illinois at Urbana-Champaign, Urbana, Illinois, United States of America; 2 Beckman Institute, University of Illinois at Urbana-Champaign, Urbana, Illinois, United States of America; 3 Department of Molecular and Integrative Physiology, University of Illinois at Urbana-Champaign, Urbana, Illinois, United States of America; Claremont Colleges, United States of America

## Abstract

Natural auditory scenes such as frog choruses consist of multiple sound sources (i.e., individual vocalizing males) producing sounds that overlap extensively in time and spectrum, often in the presence of other biotic and abiotic background noise. Detection of a signal in such environments is challenging, but it is facilitated when the noise shares common amplitude modulations across a wide frequency range, due to a phenomenon called comodulation masking release (CMR). Here, we examined how properties of the background noise, such as its bandwidth and amplitude modulation, influence the detection threshold of a target sound (pulsed amplitude modulated tones) by single neurons in the frog auditory midbrain. We found that for both modulated and unmodulated masking noise, masking was generally stronger with increasing bandwidth, but it was weakened for the widest bandwidths. Masking was less for modulated noise than for unmodulated noise for all bandwidths. However, responses were heterogeneous, and only for a subpopulation of neurons the detection of the probe was facilitated when the bandwidth of the modulated masker was increased beyond a certain bandwidth – such neurons might contribute to CMR. We observed evidence that suggests that the dips in the noise amplitude are exploited by TS neurons, and observed strong responses to target signals occurring during such dips. However, the interactions between the probe and masker responses were nonlinear, and other mechanisms, e.g., selective suppression of the response to the noise, may also be involved in the masking release.

## Introduction

To perceive the content and location of a target sound in a complex acoustic environment, the auditory input from this sound source must be grouped and separated from other sound sources in the environment [1], [2], [3]. All sounds arriving at the ears are decomposed into separate frequency-channels by the hair cells in the inner ear. To form a coherent percept of different sound sources in the environment, the information from the separate frequency channels needs to be recombined, or integrated across different frequency bands and across both ears. These processes are thought to occur in the central nervous system and grouping of acoustic streams is based on common properties across frequency bands, e.g., common sound onset, modulation, etc.

Comodulation masking release (CMR) is a process that plays a role in the analysis of auditory scenes [4], [5], [6], [7], [8]. CMR facilitates the detection of a sound source embedded in background noise when the latter has a common modulation across different frequency bands. It has been shown psychophysically in humans that for unmodulated or incoherently modulated masking noises, masking becomes more pronounced with increasing masker bandwidth up to a certain bandwidth, the critical bandwidth, beyond which signal detectability stays the same. However, for coherently modulated maskers, detectability of the target sound improves when the masker bandwidth is increased beyond the critical bandwidth, i.e., masking is released. Such a behavioral CMR-effect has also been shown in starlings [9], [10], barn owls [11] and gerbils [12].

CMR confers advantages for animals that communicate by sound in acoustically cluttered environments [13]. Many natural sounds are broadband and modulated, and most background noise is slowly modulated due to atmospheric conditions [14], [15], [16]. For numerous frog species, males congregate around a breeding pond and form a multi-species chorus. Female frogs need to detect and localize a conspecific male acoustically in order to avoid across-species breeding [17], [18]. Since a chorus has a pronounced modulation [19], we investigated whether frog central auditory neurons exhibit CMR-properties. We used the CMR-paradigm that was used in the original human psychophysics study [4] in which the masking decreased for broadband modulated noise while masking at these bandwidths remained high for unmodulated noise. The present study focuses on the torus semicircularis (TS) – a major sound processing center in the midbrain where significant across-channel integration occurs [20] – and investigates whether there is a decrease in masking for wideband modulated noise.

Neural correlates of CMR have been investigated in cats [19], [21], starlings [22], [23], [24], [25] and guinea pigs [26], [27]. At this time, the precise mechanism underlying CMR remains unclear and it is also not resolved whether CMR has a central or more peripheral origin. Several different mechanisms have been proposed for CMR [6], [28], [29], [30], [31]. Most psychophysical evidence supports the *dip-listening* mechanism [29], [32], [33] which assumes that information about the target sound is predominantly derived from signal occurring during the dips of the masker. Coherently modulated noise bands outside the target band provide information about the location of dips in the masker, and help separate the target from the masker. Other mechanisms that have been proposed are the *cross-correlation* and *equalization-cancellation* mechanisms. The cross-correlation mechanism proposes that the signals in the different channels are cross-correlated, while in the equalization-cancellation mechanism the levels of the signals in the different channels are first equalized, after which they are subtracted [31]. A second goal of this study was to examine whether there is evidence for any of these mechanisms in the frog TS.

## Materials and Methods

Extracellular recordings were made from single neurons in the TS of male Northern leopard frogs (*R. pipiens pipiens*) ranging in weight from 12 to 36 grams. Frogs were obtained from Kons Scientific (Germantown, WI) and were wild-caught in Northern Wisconsin. In the lab, frogs were kept in a temperature-controlled environment (18°C), at a 12-hour light-dark cycle. Animal care and use protocols were approved by the Laboratory Animal Care Committee of the University of Illinois at Urbana-Champaign (IACUC 03201).

### Surgical procedures

Experimental methods and procedures have been described in detail previously [34]. Briefly, the frogs were anesthetized by hypothermia [35], [36] and immobilized by intramuscular injection of *d*-tubocurarine chloride (10 µg/g body weight). The skin and underlying muscles were cut away to expose the dorsal skull. A small hole was drilled in the skull to expose the optic tectum, and a cut was made in the dura mater and pia mater above the optic tectum. The frog was then transferred to a sound attenuated chamber (IAC no. 404), of which the walls and ceiling are covered with 12″ acoustic foam wedges. During the recording session, immobilization was maintained by injection of 5 µg/g *d*-tubocurarine chloride every two hours. Frogs were covered with moist gauze to facilitate cutaneous respiration. Recordings were made from the left side of the TS, using glass microelectrodes with a tip diameter of 1–2 µm that were filled with potassium acetate in Tris buffer (0.05 M). The electrode was advanced using a remotely controlled piezoelectric microdrive (RSF Electronik). Auditory cells were found at a depth of 500–1500 µm from the surface of the midbrain. That the electrode was located in the auditory nuclei was confirmed from the usually clearly audible background responses to the search stimulus. Neurons were recorded from all regions of the TS, but predominantly from the principal and magnocellular nuclei. Action potentials were amplified using a Dagan 2400 preamplifier, bandpass filtered using Krohnhite 3700 and A-M systems 3300 filters and recorded using Tucker-Davis (TDT) system-II hardware and software running on a Microsoft-Windows PC.

### Experimental setup

TDT system-II software was used for stimulus generation and presentation. A two-channel sound delivery system was used to generate signal (‘probe’ or ‘target’) and noise (‘masker’) independently. Probe and masker were independently attenuated, and mixed on a summing amplifier (Sony GX59ES); the signal was then broadcast through a free-field loudspeaker (ADS 200LC) that was positioned on the opposite side of the recording site, at a distance of 55 cm from the frog. TDT BrainWare was used to record spike shapes and times. The spikes were sorted offline to eliminate spurious noise. Data were imported into MatLab (The Mathworks) for further analysis.

### Acoustic stimuli and experimental protocol

**Figure 1:**
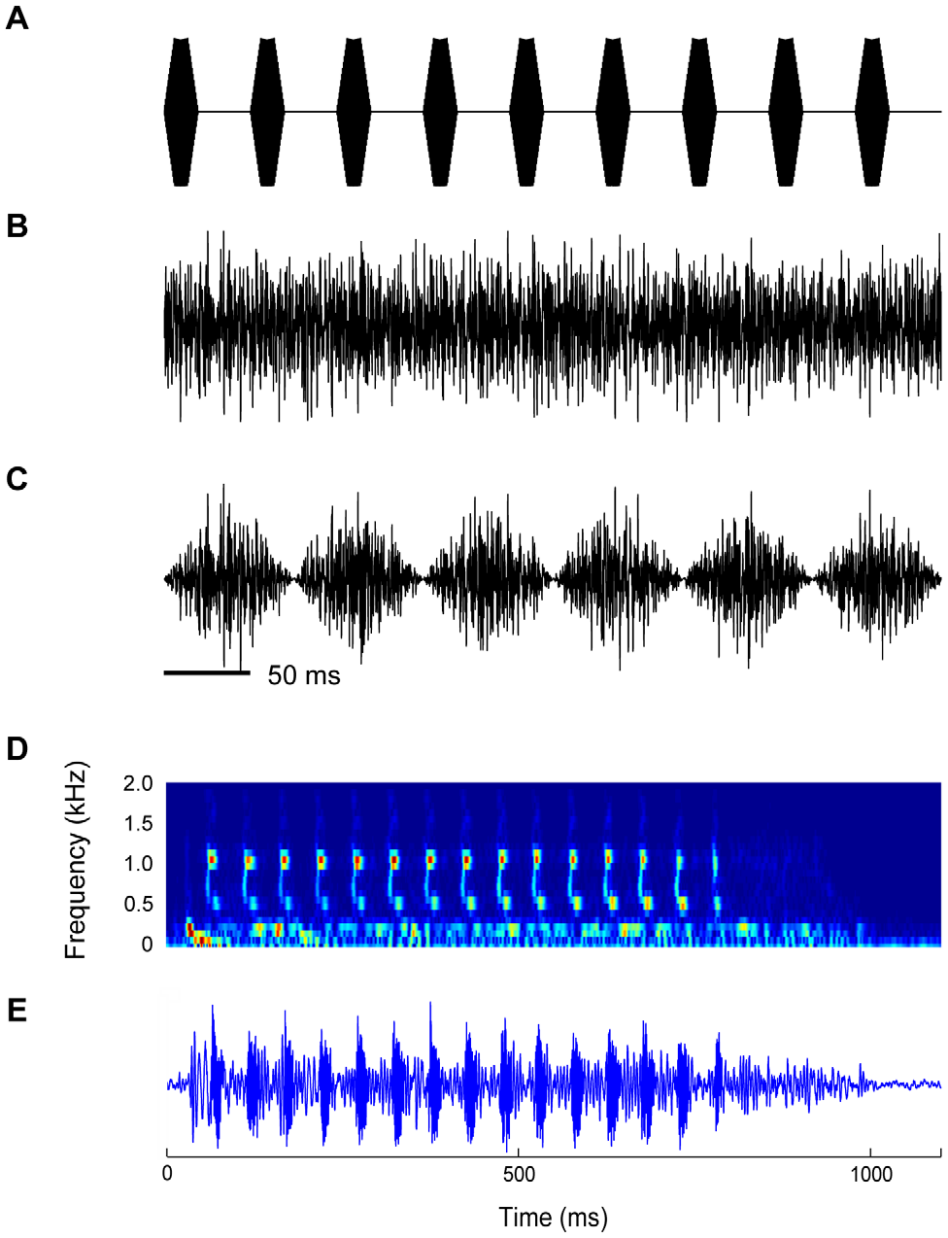
Acoustic stimuli employed in the experiments. **A:** The probe (P) was a PAM tone at 20 pulses/s with the carrier at the neuron's CF. The maskers are noises with different bandwidths centered around the neuron's CF (wideband noise shown); these were either unmodulated (M_u_, **B**) or amplitude modulated (M_m_, **C**) with a modulation depth of 100%. The rise- and fall times for M_u_ were 5 ms. Spectrogram (**D**) and waveform (**E**) of the *Rana pipiens* advertisement call.

Acoustic stimuli comprised tone bursts, noise bursts (maskers), and a probe (Fig. 1). The search stimuli were unmodulated broadband noise bursts (450 ms duration, 5 ms rise and fall time, 73 dB SPL). Given that some neurons respond only weakly to broadband noise, all cells that showed spontaneous activity or auditory responses were tested with tone bursts or pulsed amplitude modulated (PAM) tones at different frequencies or with a pre-recorded natural species mating call. After a unit was isolated, its basic response properties, i.e., its characteristic frequency (CF) and minimum threshold at CF were determined using tone bursts (450 ms duration, 5 ms linear ramp time). The probe (P) comprised a short trill of tone pulses at the unit's CF having a temporal structure similar to the species advertisement call [37]. The trill consisted of 9 tone pulses of 20 ms (including 5 ms linear ramp times) separated by an inter-pulse-interval of 30 ms (Fig. 1A) resulting in a pulse rate of 20 pulses/s. The total duration of the trill was 450 ms. The masker (M) was a 450 ms unmodulated- (Fig. 1B) or sinusoidally-amplitude-modulated (100% modulation depth; Fig. 1C) white noise of varying bandwidth centered around the unit's CF. The modulated noise was generated by multiplying the white noise with a sinusoid of 6.7 Hz. The masker level was corrected for loudspeaker characteristics. The responses to noise having different bandwidths at constant spectral level were determined. The masker bandwidths ranged from 0.1 to 2.5–5 kHz. For bandwidths >2 kHz the noise bandwidth covered the entire audible range of *R. pipiens pipiens* (∼2 kHz). The critical band for frogs ranges from 0.15–0.5 kHz for different frog species [38], [39], [40]. Since the spectrum level was constant, doubling of the noise bandwidth produces a 3 dB increase in sound level. Because CMR is normally observed at sound intensities well above threshold, the level of an unmodulated masker with a bandwidth of 1 kHz was 15 dB above the unit's threshold at CF. TS neurons typically responded to this masker level for all bandwidths. Each stimulus was presented 20 times, at an inter-stimulus interval of 2 s. Since the primary goal of this study was to determine whether and how signal detectability changes as a function of masker bandwidth and amplitude modulation, for each unit the rate-level-function (RLF) to the probe in masker (P+M) was compared to the unit's response to the masker alone (M). The paradigm was based on the psychophysical CMR-paradigm by Hall et al. [4], where the detection threshold of a probe tone was determined in the presence of modulated and unmodulated noise. The spectrum level of the masker was the same in the modulated and unmodulated case; modulated noise was coherently modulated across all frequencies with a modulation depth of 100%. Similar to the aforementioned study, in the case of a CMR the probe detection thresholds in the presence of wideband modulated maskers are expected to be lower than for narrowband maskers, and they are expected to be lower than for the unmodulated wideband noise.

### Response to masker (M) as a function of bandwidth

We determined each unit's responses to unmodulated and modulated maskers as a function of the masker bandwidth. The masker bandwidths were 0.1, 0.2, 0.4, 0.8, 1.7 and 2.5–5 kHz, the latter covering the entire audible range of *R. pipiens pipiens*. Twenty trials per bandwidth were used.

### Response to probe in masker (P+M)

To determine how a masker affected a neuron's response to the probe, its masked-RLFs (mRLFs), i.e., the RLFs to the probe in the presence of masker, were derived for both modulated and unmodulated maskers and compared to the RLF in response to the probe alone (P-alone). Masked-RLFs were collected in steps of 5 dB with 20 trials per probe level, or in steps of 2 dB and 10 trials per level. In the latter case, neighboring probe levels were averaged to ensure a consistent number of 20 trials in the analysis. For each unit, three to six mRLFs were collected for modulated and unmodulated maskers (the minimum was three mRLFs: narrow-, medium- and wideband noise for P+M_m_ and P+M_u_, if time permitted up to six bandwidths were collected for P+M_m_ and P+M_u_).

### Data analysis

The probe detection thresholds were calculated from the units' RLFs. The detection threshold for probe in masker was estimated based on the numbers of spikes recorded during the stimulus period (450 ms) in response to P+M and M-alone using the *d′*-statistic [41], [42]. *d*′ was calculated according to Sakitt [43]:
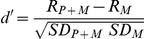
where *R_P+M_* is the spike rate in response to the probe in the presence of masker, *SD_P+M_* is the standard deviation (SD) of the response to the probe in the presence of masker, *R_M_* is the response to the masker alone, and *SD_M_* is the SD of the response to M-alone. The distributions of the spike counts were approximately Gaussian. The probe detection threshold was defined as the probe level at which *d′* = 1. For the majority of neurons, the detection threshold could be determined using this method.

**Figure 2:**
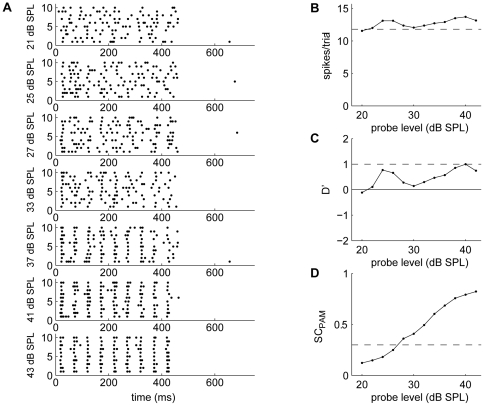
Example of a neuron that shows overt time locking to the probe. The unit's responses to P+M_u_ at increasing probe level (**A**) show firing periodicity corresponding to the envelope of the probe. The number of spikes remained approximately constant for increasing probe levels (**B**, dashed line indicates response to noise alone) and *d*′ did not reach threshold, and thus the probe is not detectable based on the *d*′-method (**C**). However, the probe signal is clearly detectable based on the SC (**D**).

For some neurons, increasing the probe level produced little or no increase in spike count, and the criterion of *d*′ = 1 was not reached, although the neuron exhibited overt time-locked discharges to the probe, as also observed in the gerbil cochlear nucleus [44]. Figure 2 shows an example of such a neuron. To determine the degree of envelope-following to the probe we computed the synchronization coefficient (SC) according to Goldberg and Brown [45]:
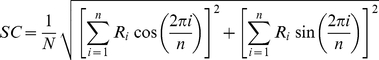
where *N* is the total number of spikes in the period histogram, *n* is the number of bins (binsize 1 ms), and *R_i_* is the number of spikes in bin *i*. A Rayleigh test of uniformity was performed to determine whether the directionality of the response was significant. Only significant SCs were used and the detection criterion for the SC was SC = 0.3.

In cases of overt time locking in the absence of an increase in spike rate, the SC was used to determine the detection threshold. Because probe and modulated maskers had different periodicities, the firing synchronization to the probe and masker could be used to gain insight into the CMR mechanism. The advantages of using the SC were: 1) the probe could be detected even when units did not show an increase in spike count at increasing probe levels, 2) the SC was more robust, 3) the SC was often more sensitive than the *d′*-method. The disadvantage is that the SC can only be used for neurons that exhibit time-locked firing. The SC as a measure of detectability of a probe in masker was used previously [44], [46], [47], [48] and was shown to yield lower detection thresholds than a rate-based metric [47]. However, many neurons did not show time-locked responses to the probe, and since there is no equivalence between detection criteria based on SC and *d*′, the SC was used only if the *d*′-method failed to produce an estimate of the detection threshold, and the SC was only used in relative metrics, e.g., characterization of the shape of the curve of detection threshold versus bandwidth.

Many properties of the neurons were not normally distributed. Therefore, data were characterized by their medians. For statistical testing a Wilcoxon paired signed rank test was used. Categorical data were tested for marginal homogeneity using Bhapkar and McNemar tests.

## Results

Responses to masker as a function of its bandwidth were obtained from 166 TS neurons from 73 frogs. For 115 of the 166 neurons, we obtained responses to the probe in the presence of masker (P+M). The neural responses to tone bursts and P-alone were described previously [34]. Briefly, neuronal CFs ranged from 0.1–1.8 kHz and were clustered around 0.1–0.5 kHz, 0.7–1.2 kHz, and 1.5 kHz, in agreement with earlier studies in *Rana pipiens pipiens* [20], [49], [50]. In response to tone bursts at the unit's CF, TS neurons exhibited either a phasic (31%) or tonic (69%) discharge pattern, using the classification scheme of Gooler and Feng [49]. Detection thresholds for P-alone ranged from 11–87 dB SPL but fell largely within a band of 20–40 dB SPL, with a median of 32.5 dB SPL with interquartile range (*iqr*) of 14.9 dB SPL.

### Effect of masker on spike rate and temporal response pattern

**Figure 3:**
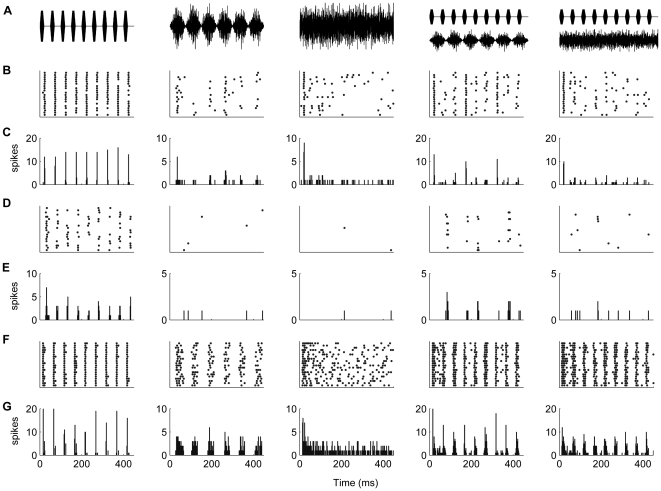
Firing patterns of different neurons. Rasterplots and peristimulus time histograms (PSTHs) showing the temporal firing patterns to P-alone (1^st^ column), wideband M_m_ (2^nd^ column), wideband M_u_ (3^nd^ column), P+M_m_ (4^th^ column) and P+M_u_ (5^th^ column) for three representative neurons (**B–G**). The stimuli are shown in (**A**). The binsize for the PSTHs was 2 ms. For the neuron in (**B**, **C**) and (**D, E**) addition of the masker degrades the temporal pattern of the response to the probe. In panels **B** and **C**, the probe responses occurring during the dips of the M_m_ are unaffected (1^st^, 4^th^ and 7^th^ pulse in the 4^th^ column), whereas the unit's firing rate and time-locking to the probe are decreased when the probe coincides with the masker. For the neuron in (**D, E**) the presence of the masker severely disrupted the response to the probe. In the presence of modulated noise, only the response to the 2^nd^, 5^th^, and 8^th^ pulse remained (4^th^ column). The neuron in (**F, G**) responds to the temporal pattern of the probe despite the presence of maskers albeit the detection thresholds were elevated. In the presence of modulated masker, timing accuracy to the pulses occurring in the dips (1^st^, 4^th^ and 7^th^ pulse, 4^th^ column) was higher than to pulses occurring during other phases.

The presence of masker generally altered a unit's spike rate and temporal discharge pattern to the probe. Figure 3 shows the responses of three neurons that showed periodic discharges to the probe (left column). The responses to P+M indicate that the presence of masker disrupted the regularity of the response to the probe (4^th^ and 5^th^ column). Figure 3B and C show the disruption of the regular firing pattern to the probe by M_m,_ M_m_ disrupted the response during the peaks of the masker, but left the response during the dips (1^st^, 4^th^ and 7^th^ probe tone) more or less unaffected. Figure 3D–E shows drastic suppression of the unit's probe response by M_u_ although the unit did not respond to M_u_ alone. The modulated masker showed less severe suppression and the response to the 2^nd^, 5^th^ and 8^th^ probe tone were retained. In Fig. 3F, the overall spike rate is increased for P+M, however when P coincided with M_u_ or the peaks of M_m_ the neuron's temporal precision was degraded (Fig. 3G).

66 neurons had either time-locked responses or onset responses to the probe. In many cases the responses to P and M_m_ were additive and the spike rate in response to P+M_m_ was highest when pulses of the probe coincided with the peaks of M_m_ (Fig. 3F–G, 29 neurons). In other cases, adding M_m_ suppressed a unit's probe response during the peak of the masker, leaving the response during the dip (Fig. 3B–C, 6 neurons) or the falling phase (Fig. 3D–E, 5 neurons) of the masker unchanged or minimally affected. A few cases (3 neurons) could not be classified, and the remaining neurons showed an equal response to all probe pulses after adding M_m_. For neurons that did not show time-locked responses to the probe adding a masker did not lead to noticeable changes to the temporal discharge pattern to the probe.

### Bandwidth dependence of the response to M-alone

**Figure 4:**
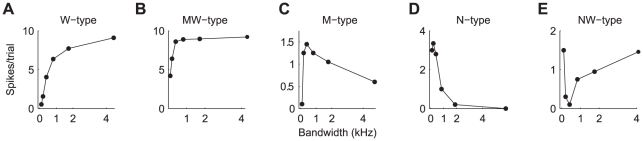
Representative examples of the different types of responses to M_m_ as a function of bandwidth. **A:** monotonically increasing (W-type). **B:** monotonically increasing response that saturates (MW-type). **C:** M-type neuron showing an initial increase followed by a decrease. **D:** Decreasing response (N-type). **E:** initial decrease in response followed by an increase (NW-type).

Without exception, the response of TS neurons to M-alone depended on the masker bandwidth. For 166 neurons, the response to modulated masker was acquired, and for 109 neurons the responses to both masker types was acquired. Five different types of response functions could be distinguished (Fig. 4). Assignment to a given response-type was determined by the difference between the narrowest-, middle- and widest bandwidths; an increase or decrease in spike rate of 5% determined the difference between M-, W- and MW-type, and between N- and NW-type. The label indicates the bandwidth to which the neuron is most responsive, i.e., narrow, medium or wide. The different response types reflect differential across-frequency integration or inhibition: 1) W-type neurons showing a monotonically increasing response to an increase in masker bandwidth (Fig. 4A). Such neurons responded most strongly to wideband masker, and least to narrowband masker. This response type was observed for 13% (14/109) of the neurons in response to modulated masker (M_m_), and 9% (10/109) in response to unmodulated masker (M_u_). 2) MW-type neurons showing a rapid increase in their response with increasing bandwidth, after which the response saturated (Fig. 4B, 15% for M_m_ (16/109), and 16% (17/109 for M_u_)). 3) M-type neurons were characterized by an initial increase in spike rate followed by a decrease for wideband noise (Fig. 4C, 45% for M_m_ (49/109), and 42% (46/109 for M_u_)); these neurons thus showed a response maximum for maskers of intermediate bandwidths. 4) N-type neurons responded most strongly to narrowband masker and showed a progressive decrease in response with increasing bandwidth (Fig. 4D, 17% for M_m_ (19/109), and 27% (29/109 for M_u_)). 5) NW-type neurons showed an initial decrease followed by a subsequent increase in response as the masker bandwidth was increased (Fig. 4E, 4% for M_m_ (4/109), and 4% (4/109 for M_u_)); these neurons showed a response minimum for maskers of intermediate bandwidths. Only a few neurons showed responses that could not be classified into one of these categories (O-type, 6% for M_m_ (6/109), and 3% (3/109 for M_u_)).

### Differences between modulated and unmodulated maskers

**Figure 5:**
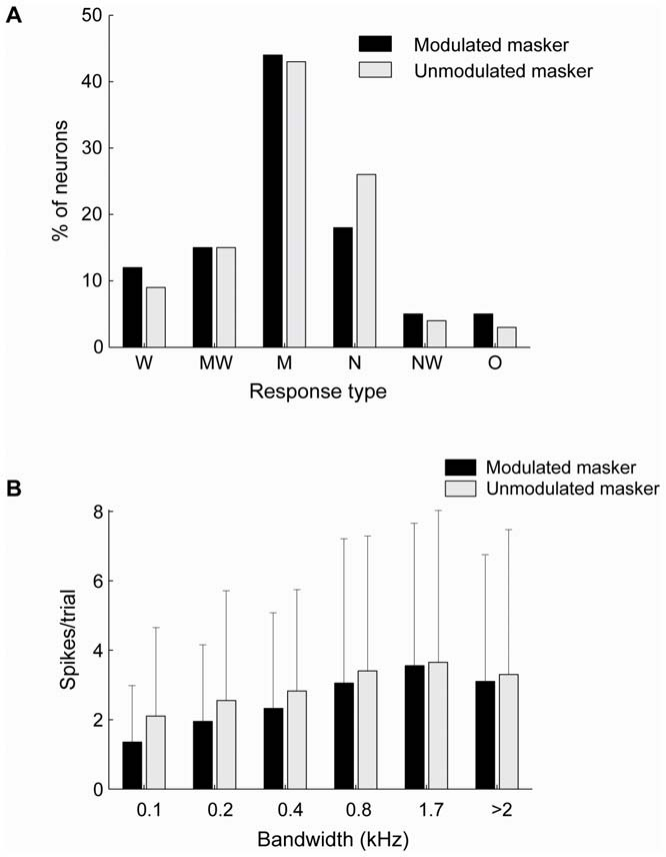
Population characteristics of the responses to M_m_ and M_u_. **A:** Relative occurrence of the different types of response functions (see Fig. 4) for M_m_ (black) and M_u_ (gray). **B:** Median response over the population of neurons to M_u_ (gray) and M_m_ (black) having different bandwidths. Included are data from 109 neurons for which responses to both M_m_ and M_u_ were acquired. Errorbars indicate interquartile ranges; the large interquartile ranges illustrate the high variability of the neural responses.

For the 109 TS neurons for which responses to both modulated and unmodulated maskers were acquired, the shape of the unit's response function was the same in 69% of the cases; the remaining neurons displayed differential response functions. For both modulated and unmodulated maskers (Fig. 5A), the M-type was the most common response pattern, with the NW-type being the least common. The W-type (monotonically increasing response pattern) was observed slightly more frequently for modulated maskers, while the N-type, with monotonically decreasing response pattern, was more prevalent for unmodulated maskers. There were significant differences in the shapes of the response curves for modulated and unmodulated maskers (*p* = 0.02, Bhapkar test). The N-type category showed significant differences in row and column proportions (*p* = 0.01, McNemar test, Bonferroni-corrected).

Figure 5B shows the median firing rates for modulated and unmodulated maskers having different bandwidths (at equal peak noise level). For both maskers, the median spike count of the population of neurons increased progressively with increasing masker bandwidth, but decreased for the widest bandwidth. The decrease between 1.7 kHz and >2 kHz was significant, as were all increases in neighboring bandwidths except between 0.8 kHz and 1.7 kHz (*p*<0.05, Wilcoxon signed rank test, Bonferroni corrected for multiple comparisons). There was a significant difference in average spike count between the two types of masker when the masker bandwidth was ≤0.4 kHz (0.1 kHz: *p* = 10^−13^, 0.2 kHz: *p* = 10^−8^, 0.4 kHz: *p* = 0.0002; Wilcoxon signed rank test, 109 neurons). The differences were not significant for masker bandwidths >0.4 kHz (0.8 kHz: *p* = 0.60, 1.7 kHz: *p* = 0.11, >2 kHz: *p* = 0.26; Wilcoxon signed rank test, 109 neurons). The median difference in spike rate between M_m_ and M_u_ ranged from 0.05–0.65 spikes per trial: the median M_u_-M_m_ was 0.65 spikes/trial for a bandwidth of 0.1 kHz, 0.5 spikes/trial for 0.2 kHz, 0.28 spikes/trial for 0.4 kHz, 0.05 spikes/trial for 0.8 kHz, 0.30 spikes/trial for 1.7 kHz and 0.11 spikes/trial for >2 kHz. Thus, differences in response between M_u_ and M_m_ decreased with bandwidth and were minor for bandwidths >0.4 kHz.

### Response to probe in masker as a function of masker bandwidth

**Figure 6:**
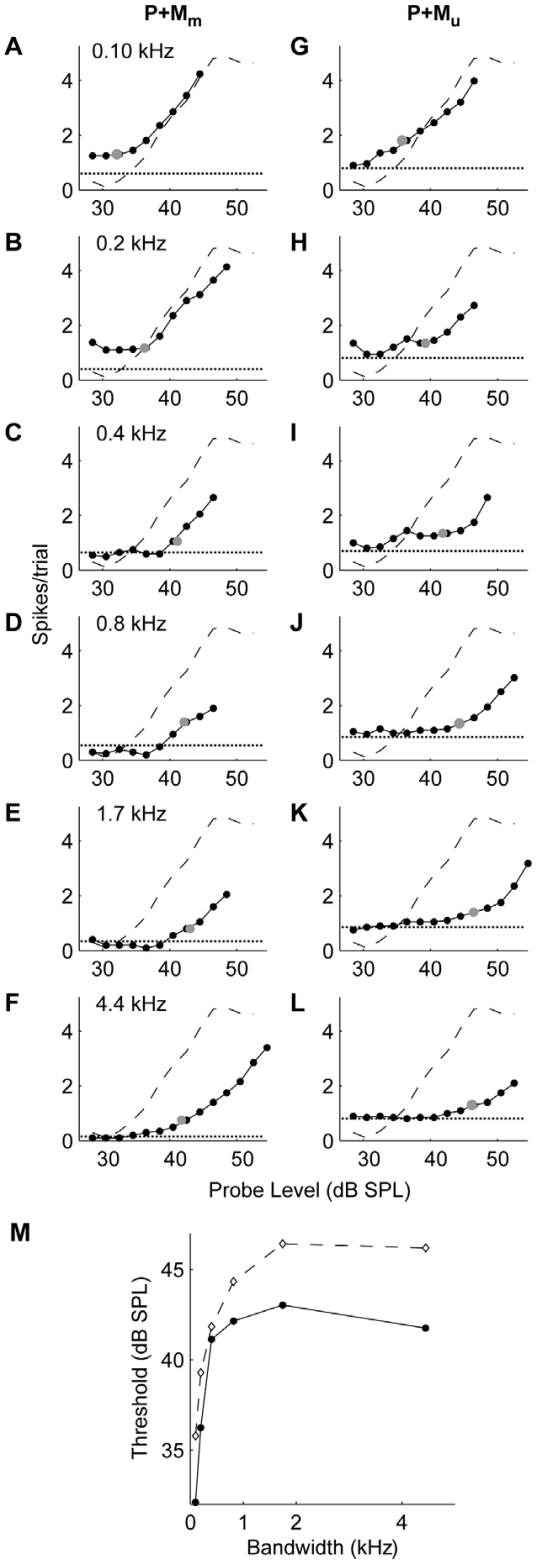
Effect of masker bandwidth on probe detection threshold. **A–F:** RLFs for M_m_; the dashed line indicates the RLF for the probe alone (P) and the solid line shows the mRLF for the probe in the presence of masker, while the dotted line shows the response to masker alone. The masked threshold is indicated by the gray dots. **G–L:** RLFs in response to M_u_. **M:** detection thresholds as a function of the masker bandwidth for P+M_u_ (dashed line) and P+M_m_ (solid line).

Figure 6 shows the RLF of a representative neuron's response to P-alone (dashed curve in columns 1 and 2), and its mRLFs to P+M for modulated (M_m_) and unmodulated maskers (M_u_) (solid curves in columns 1 and 2, respectively). For both maskers, an increase in bandwidth shifted the mRLF progressively to the right, indicating increasing suppression of the probe response. The probe detection threshold (Fig. 6M) was calculated from the unit's responses to P+M and to M-alone (dotted line). For M_u_ (dashed line in Fig. 6M), this neuron showed a progressive elevation of the probe detection threshold (i.e., increased masking) with increasing masker bandwidth, reaching saturation at a bandwidth of 2 kHz. For M_m_ (solid line in Fig. 6M), masking was lower, likely due to the lower RMS noise level of M_m_ and here an increase in masker bandwidth initially elevated the unit's probe detection threshold (up to 2 kHz), but a further increase in bandwidth resulted in release from masking.

**Figure 7:**
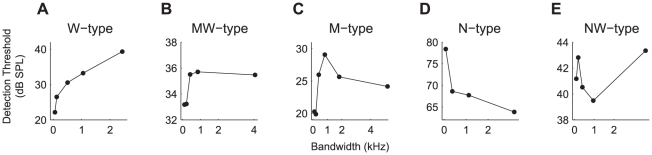
Differential effects of masker bandwidth on probe detection threshold for M_m_. The different response types that were observed were: **A:** a monotonic increase in probe detection threshold for increasing noise bandwidths (W-type neurons). **B:** a monotonic increase in detection threshold that levels off (MW-type), **C:** an initial increase followed by a decrease in threshold at increasing bandwidths (M-type), **D:** a systematic decrease in detection threshold with increasing bandwidth (N-type). **E**: an initial decrease followed by an increase in threshold at increasing bandwidths (NW-type).

The relationship between the detection threshold and bandwidth of M_m_ was variable (Fig. 7). The following response curves as a function of masker bandwidth were observed: 1) W-type that was characterized by a monotonic increasing probe detection threshold with increasing masker bandwidth (Fig. 7A), 2) MW-type, featuring a rapid increase in detection threshold that leveled off with further increases in bandwidth (Fig. 7B), 3) M-type, which featured the strongest masking (i.e. highest probe detection threshold) at an intermediate masker bandwidth (Fig. 7C), thereby showing masking release with an increase in bandwidth, 4) N-type that showed maximal masking by narrowband noise, and progressively less masking by medium- and wideband noise (Fig. 7D), and thus also showed masking release with increasing masker bandwidth, 5) NW-type that showed initially reduced masking, followed by an increase in probe detection threshold with increasing masker bandwidth (Fig. 7E); these neurons showed maximum masking release for an intermediate masker bandwidth. Neurons were assigned to a category based on the difference between the middle bandwidth and the narrowest and widest bandwidth, increases or decreases of >1 dB SPL determined allocation to M-type vs. W-type and MW-type, and N-type vs. NW-type.

### Masking of probe in M_m_ and M_u_

**Figure 8:**
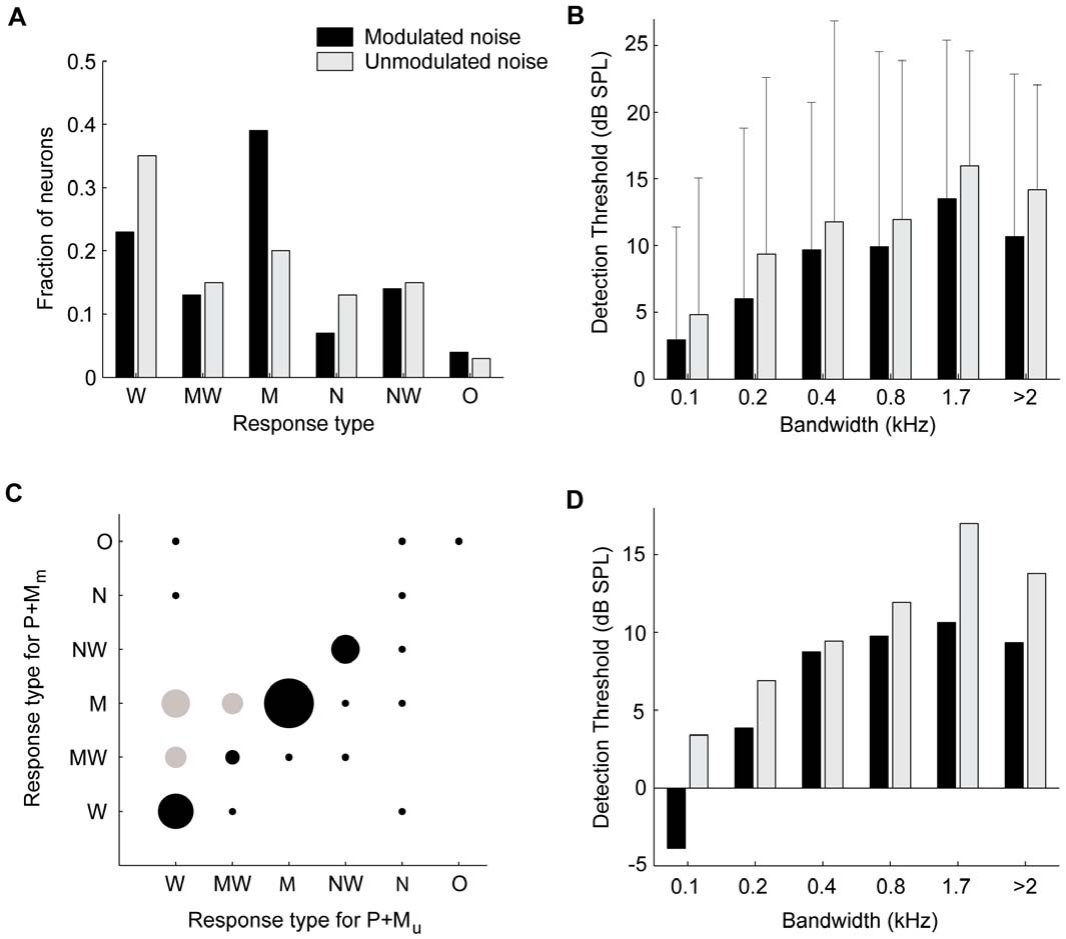
Population characteristics of the responses to P+M_m_ and P+M_u_. **A:** Distribution of the different response functions (see Fig. 7) for P+M_m_ (black) and P+M_u_ (gray). ‘O’ indicates other types of responses. **B:** Median threshold increase (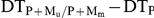) for M_m_ (black) and M_u_ (gray), as a function of masker bandwidth. Errorbars indicate interquartile ranges; large interquartile ranges point to the high variability in the neural responses. **C:** Relative occurrence of the different types of threshold curves for P+M_m_ (ordinate) and P+M_u_ (abscissa). The size of the dots indicates the percentage of neurons, with the largest dot corresponding to 17.5%. Combinations that can lead to CMR-type behavior are indicated in gray. **D:** Median detection thresholds for M_m_ (black) and M_u_ (gray) for the off-diagonal neurons (gray circles) in **C**.

The distribution of the response-types to P+M_m_ and P+M_u_ is shown in Fig. 8A; it represents all neurons for which masking curves in response to P+M_m_ as well as P+M_u_ were acquired. Most neurons (72%) showed the same type of response curve for M_m_ and M_u_. For M_u_ the W-type (monotonically increasing function of masker bandwidth) was the most common (35%); other response functions were less common (13–20% of neurons each). In contrast, for M_m_ the M-type response function (39%), showing release from masking for wideband maskers, was most prevalent. For most TS neurons, the presence of a masker elevated the unit's probe detection threshold. The median increases in threshold as a function of masker bandwidth are shown in Fig. 8B for M_m_ and M_u_. Figure 8B includes all neurons for which the responses to both P+M_m_ and P+M_u_ were acquired for a given bandwidth. For both maskers, the median probe detection thresholds increased progressively with increasing masker bandwidth; at a masker bandwidth of 0.1 kHz the increase in probe detection threshold was about 4 dB SPL, which increased to 13–16 dB SPL at a bandwidth of 1.7 kHz, and decreased slightly at bandwidths >2 kHz. 25% of TS neurons showed a decrease in detection threshold when a masker was present, i.e., a facilitating effect, but this was observed primarily with narrowband modulated maskers. Detection thresholds were significantly lower for P+M_m_ than for P+M_u_ for bandwidths of 0.1 kHz, 0.2 kHz and >2 kHz (*p* = 0.002 for 0.1 kHz (27 neurons), *p* = 0.004 for 0.2 kHz (53 neurons), *p* = 0.07 for 0.4 kHz (42 neurons), *p* = 0.17 for 0.8 kHz (24 neurons), *p* = 0.64 for 1.7 kHz (21 neurons) and *p* = 0.002 for bandwidths >2 kHz (55 neurons), Wilcoxon signed rank test). The median difference in threshold elevation between P+M_m_ and P+M_u_ ranged from 0–2.1 dB SPL (the median was 2.1 dB SPL for a bandwidth of 0.1 kHz, 1.9 dB SPL for 0.2 kHz, 0 dB SPL for 0.4 kHz, 0.3 dB SPL for 0.8 kHz, 0 dB SPL for 1.7 kHz and 1.2 dB SPL for >2 kHz). Thus, across the population the detection thresholds for P+M_m_ were significantly lower than for P+M_u_ for bandwidths of ≤0.2 kHz and >2 kHz. The behavioral CMR is characterized by distinctly different curves for the threshold as a function of bandwidth for the two types of maskers [4]. However, across the population of TS neurons the detection thresholds were lower only for bandwidths >2 kHz, as well as for narrowband M_m_.

If the behavior is subserved by a subpopulation of neurons, such neurons are expected to have different response curves for P+M_m_ vs. P+M_u_. The distribution of the shapes of the masking response curve for P+M_m_ vs. P+M_u_ is shown in Fig. 8C. On the diagonal are neurons that showed the same type of masking response curves for M_m_ and M_u_. There were significant differences in the curve shapes for the different masker types (*p* = 0.009, Bhapkar test). The W- and M-type categories showed significant differences in row and column proportions (*p* = 0.03 and *p* = 0.01 respectively, McNemar test, Bonferroni-corrected). About 15% of TS neurons (gray circles) belonged to this off-diagonal category displaying W- or MW-type masking response curves for M_u_, and MW- or M-type for M_m_. Figure 8D shows the median threshold elevation for P+M as a function of bandwidth for these off-diagonal neurons. However, this is a subpopulation, consisting of relatively few neurons, and the difference between modulated and unmodulated maskers reached significance only for the widest bandwidth (>2 kHz, *p* = 0.009, Wilcoxon signed rank test, 8 neurons) with a median difference of 4.0 dB SPL.

## Discussion

In this study we investigated how masker bandwidth and amplitude modulation affect masking of a PAM tones with a similar temporal structure as the frog mating call. We found that the degree of masking depended on the masker bandwidth and its modulation, i.e., M_m_ was a less potent masker compared to M_u_ for narrowband and wideband maskers, but not for medium-band maskers. Across the population masking increased with increasing bandwidth, but decreased for the widest bandwidths.

### Response to M-alone

The neural responses to M-alone were bandwidth-dependent for all neurons. The median response of the population increased with bandwidth, reflecting an increase in energy as the bandwidth increases, although for the widest bandwidth the response decreased. Klump and Nieder [22] observed similar responses to noise in the starling forebrain. The stronger response of the neural population to wideband masker was also shown using fMRI in the human inferior colliculus (IC), where the functional activation increased with bandwidth for unmodulated masker both at constant spectrum level and at constant sound pressure level [51]. Here, we kept the spectrum level constant, in keeping with the psychophysical CMR-paradigm [4], and band-widening paradigms used in other studies [22], [52], [53]. In some studies, the sound pressure level was kept constant [54]; a constant sound pressure level however, leads to a decrease in spectrum level with increasing bandwidth, and may confound bandwidth-dependent releases from masking.

Effects of changing masker bandwidth were heterogeneous. Some neurons showed an increase in response to the masker as its bandwidth increased and were thus most responsive to wideband noise; other neurons responded mainly to narrowband noise, or were most responsive to maskers having intermediate bandwidths. A variety of response curves, similar to the ones observed here, was also observed in the mammalian cochlear nucleus [52], [53], [55], [56] and monkey auditory cortex [54]. A possible biological significance of bandwidth-selectivity is that it confers sensitivity to different types of environmental sounds, e.g., vocal signals [13]. Differences in neural frequency-tuning may underlie these differences in curve shapes. However, differences in curve shapes between M_m_ and M_u_ that were observed in 31% of frog TS neurons cannot easily be explained by frequency tuning, and indicate a complex interaction between bandwidth and amplitude modulation, i.e., their effects on the neural response are not independent. Neurons that show differential bandwidth-dependence when sounds are modulated could be more sensitive to specific vocalizations. The differences may be caused by non-linearities in the tuning properties of neurons, such as differences in frequency tuning to modulated and unmodulated sounds [57], [58]. For instance in the cat IC [59] and in auditory cortex [60], [61] the excitatory frequency tuning curve for tones was found to deviate substantially from the tuning curve for complex sounds. To fully quantify the relationship between frequency selectivity and the response to noise of variable bandwidth, a systematic study of excitatory tuning properties, inhibitory sidebands, and tuning to noise or complex stimuli is needed.

Across the population, responses to M_m_ were lower than to M_u_, presumably due to the lower SPL of M_m_. However, the difference between M_m_ and M_u_ was not significant for bandwidths >0.4 kHz, despite the higher RMS sound level of the unmodulated masker. Also, the increase in spike rate with bandwidth was not significant from 800 to 1.7 kHz and decreased thereafter. This suggests a relative insensitivity to the RMS sound level at wide bandwidths and possible suppression for large bandwidths. The similar response to M_m_ and M_u_ at wide bandwidths despite the lower RMS sound level of M_m_ may reflect a higher sensitivity of the neurons to modulated than to unmodulated sounds. Environmental sounds are often broadband [13] and amplitude modulated [15], [62], including the advertisement calls of *R. pipiens*, and large choruses [19]. The finding that a fraction of the TS neurons responded preferentially to narrowband unmodulated noise (Fig. 5A) raises the question whether this is also an adaptation to the frog's natural environment. To determine whether this is the case, one needs to carry out a systematic recording of the bandwidth and modulation spectrum of sounds in the frog's natural environment.

### Response to probe in masker

For all neurons, we found that masker bandwidth affected the strength of the neuron's response to the probe when the masker was present. Adding a masker shifted the unit's RLF for most neurons (compared to the RLF for P-alone) and thus changed the detection threshold; shifts of the RLF to the right as well as to the left were observed. The detection threshold for the probe usually increased when a masker was present. However, shifts of the RLF to the left typically led to a lower detection threshold compared to P-alone (facilitation). For the population, the median detection threshold for probe in masker (M_m_ or M_u_) was elevated with increasing masker bandwidth. However, individual neurons show considerable variability, as indicated by the large interquartile ranges of the population, ranging from a decrease in detection threshold (i.e. facilitation) by as much as 12 dB SPL to a threshold increase of >20 dB SPL.

M_u_ and M_m_ produced significantly different masking. In general, whereas M_m_ and M_u_ both elevated the unit's probe detection threshold, M_u_ typically was a more effective masker than M_m_. This was also observed in the starling forebrain [22]. The reduced potency of M_m_ could be due to the occurrence of dips in the masker, or due to its lower SPL. However, the masker detection thresholds were not significantly different for medium band maskers, making it less likely that it is purely due to the difference in SPL. Similarly, neural responses to the masker alone were not significantly different for large bandwidths. Masking by M_m_ was lower than by M_u_ for narrowband noise and for the widest bandwidth, indicating a release from masking at the broadest bandwidth. This may lead to a possible CMR in frogs, although the masking curve shape is different from the masking curves observed in humans [4]. However, the frog's auditory range is much smaller than humans, and thus their masking curves might be very different.

Differential masking between M_m_ and M_u_ was bandwidth dependent, suggesting across-channel frequency processing. About 30% of the neurons showed different masking curves for M_m_ and M_u_ and significant changes in the types of response curves were observed between modulated and unmodulated maskers. The larger number of W-type response curves and smaller number of M-type response curves for M_u_ implies that wideband M_u_ is a more potent masker than wideband M_m_. The shape of the response curve to M-alone was similar to the shape of the masking curve in only 30% of the neurons. For instance, a strong response to the masker does not necessarily mean that the detection threshold for the probe will be elevated. Thus, the response to M-alone could not always predict masking efficacy.

Some neurons showed release from masking that was only bandwidth-dependent (like N- or M-type neurons), while others showed lower masking for M_m_ which was the same across all bandwidths. This suggests that the contributions of bandwidth and modulation to masking release may occur separately in different populations of neurons. Neurons that exhibit features like modulation-dependent release from masking or bandwidth-dependent release from masking may be precursors of a neural CMR phenomenon. A postsynaptic neuron receiving input from both types of neurons might show an effect of bandwidth *and* modulation or a CMR effect. Fifteen percent of TS neurons exhibited both bandwidth- and modulation-dependent release from masking (Fig. 8C, D) – these may contribute to a behavioral CMR. That such properties are observed in only a small population of midbrain neurons and thus do not exert marked effects on the population response, is in agreement with previous reports in other animals [23], [24], [26], [27]. Different neurons may serve different tasks, and the properties of single neurons may be combined at a higher level to give rise to a behavior [27], [63], although at the population level no clear effect is apparent.

A behavioral CMR-effect has been demonstrated in a number of species although not yet in frogs. The differences in detection threshold found here were not large, but they may be behaviorally relevant. Sound levels found in spatial release from masking in frogs were 3 dB SPL or less in green treefrogs (*Hyla cinerea*) [64], while Bee [65] found a spatial release of 6–12 dB SPL in grey treefrogs (*Hyla chrysoscelis*). He also found that spatial release from masking was most effective for a SNR difference of 0–6 dB SPL between target and masker [66]. Although we did not find an obvious correlate of the psychophysical masking curve, the response properties of a small population of TS-neurons suggest that *R. pipiens* might exhibit a behavioral CMR. However, to fully resolve whether frogs have CMR needs to be demonstrated behaviorally.

### Mechanism

The temporal pattern of a unit's response to P+M can shed light on how a masked probe is detected and perhaps on a mechanism for CMR. Although the number of neurons that showed different temporal patterns was too low to clearly correlate temporal patterns with masking curves, the temporal patterns observed for P+M_m_ can give hints about possible mechanisms. Ten percent of frog TS neurons showed evidence supporting dip-listening (Fig. 3); these neurons have strong responses to the probe when it occurs in dips of the masker, while the responses during other phases of the masker are degraded. Such neurons tend to show less masking for M_m_, and thus may be involved in modulation-dependent release from masking. In the mammalian cochlear nucleus [26], [27] flanking bands inhibited the on-frequency masker, making the probe more salient. Suppression of the response by the wideband masker was also seen in some neurons (Fig. 3), and such a mechanism might be present in neurons showing dip-listening.

Other mechanisms, such as noise suppression or compression of the envelope in the auditory periphery [19], [31], [67] may also play a role. For example, for a number of neurons, the probe overtly suppressed the unit's response to the masker (Fig. 3). Strong suppression of the response to the masker at high probe levels has also been reported by Las et al. [21] in the cat IC. For some neurons in the frog TS, however, suppression of the masker response occurred at low as well as high probe levels. The responses to the probe and to masker generally interacted non-linearly, and the response to P+M cannot be predicted on the basis of the responses to P-alone and M-alone. The heterogeneity of TS neurons and the fact that many TS neurons do not show time-locking [34] mean that a detailed study of the units' temporal responses requires a very large sample size. Further studies are needed to fully characterize the units' temporal responses, and to determine the specific masker features that contribute to release from masking.

## References

[1] Feng AS, Ratnam R (2000). Neural basis of hearing in real-world situations.. Annu Rev Psychol.

[2] Bregman AS (1990). Auditory scene analysis.

[3] Bee MA, Micheyl C (2008). The cocktail party problem: what is it? How can it be solved? And why should animal behaviorists study it?. J Comp Psychol.

[4] Hall JW, Haggard MP, Fernandes MA (1984). Detection in noise by spectro-temporal pattern-analysis.. J Acoust Soc Am.

[5] Hall JW, Grose JH (1988). Comodulation masking release - Evidence for multiple cues.. J Acoust Soc Am.

[6] Buus S (1985). Release from masking caused by envelope fluctuations.. J Acoust Soc Am.

[7] Mcfadden D (1986). Comodulation masking release - Effects of varying the level, duration, and time-delay of the cue band.. J Acoust Soc Am.

[8] Schooneveldt GP, Moore BC (1987). Comodulation masking release (CMR): effects of signal frequency, flanking-band frequency, masker bandwidth, flanking-band level, and monotic versus dichotic presentation of the flanking band.. J Acoust Soc Am.

[9] Klump GM, Langemann U (1995). Comodulation masking release in a songbird.. Hear Res.

[10] Langemann U, Klump GM (2001). Signal detection in amplitude-modulated maskers. I. Behavioural auditory thresholds in a songbird.. Eur J Neurosci.

[11] Langemann U, Zokoll MA, Klump GM (2005). Analysis of spectral shape in the barn owl auditory system.. J Comp Physiol A.

[12] Gleich O, Kittel MC, Klump GM, Strutz J (2007). Temporal integration in the gerbil: the effects of age, hearing loss and temporally unmodulated and modulated speech-like masker noises.. Hear Res.

[13] Lewicki MS (2002). Efficient coding of natural sounds.. Nature Neurosci.

[14] Morton ES (1975). Ecological sources of selection on avian sounds.. American Naturalist.

[15] Richards DG, Wiley RH (1980). Reverberations and amplitude fluctuations in the propagation of sound in a forest - Implications for animal communication.. American Naturalist.

[16] Narins PM, Zelick R, Fritz B, Ryan MJ, Wilczynski W, Hetherington TE, Walkowiak W (1988). Effects of noise on auditory processing and behavior.. The evolution of the amphibian auditory system.

[17] Blair WF (1964). Isolating mechanisms and interspecies interactions in anuran amphibians.. Quart Rev Biol.

[18] Frost JS, Platz JE (1983). Comparative-assessment of modes of reproductive isolation among 4 species of leopard frogs (*Rana pipiens* complex).. Evolution.

[19] Nelken I, Rotman Y, Bar Yosef O (1999). Responses of auditory-cortex neurons to structural features of natural sounds.. Nature.

[20] Fuzessery ZM, Feng AS (1982). Frequency-selectivity in the anuran auditory midbrain - single unit responses to single and multiple tone stimulation.. J Comp Physiol.

[21] Las L, Stern EA, Nelken I (2005). Representation of tone in fluctuating maskers in the ascending auditory system.. J Neurosci.

[22] Klump GM, Nieder A (2001). Release from masking in fluctuating background noise in a songbird's auditory forebrain.. Neuroreport.

[23] Nieder A, Klump GM (2001). Signal detection in amplitude-modulated maskers. II. Processing in the songbird's auditory forebrain.. Eur J Neurosci.

[24] Hofer SB, Klump GM (2003). Within- and across-channel processing in auditory masking: a physiological study in the songbird forebrain.. J Neurosci.

[25] Bee MA, Buschermohle M, Klump GM (2007). Detecting modulated signals in modulated noise: (II) neural thresholds in the songbird forebrain.. Eur J Neurosci.

[26] Pressnitzer D, Meddis R, Delahaye R, Winter IM (2001). Physiological correlates of comodulation masking release in the mammalian ventral cochlear nucleus.. J Neurosci.

[27] Neuert V, Verhey JL, Winter IM (2004). Responses of dorsal cochlear nucleus neurons to signals in the presence of modulated maskers.. J Neurosci.

[28] Verhey JL, Pressnitzer D, Winter IM (2003). The psychophysics and physiology of comodulation masking release.. Exp Brain Res.

[29] Buus S, Zhang LJ, Florentine M (1996). Stimulus-driven, time-varying weights for comodulation masking release.. J Acoust Soc Am.

[30] Richards VM (1987). Monaural envelope correlation perception.. J Acoust Soc Am.

[31] Buschermohle M, Verhey JL, Feudel U, Freund JA (2007). The role of the auditory periphery in comodulation detection difference and comodulation masking release.. Biol Cybern.

[32] Hall JW, Grose JH (1991). Relative Contributions of Envelope Maxima and Minima to Comodulation Masking Release.. Quart J Exp Psychol.

[33] Fantini DA (1991). The processing of envelope information in comodulation masking release (CMR) and envelope discrimination.. J Acoust Soc Am.

[34] Goense JBM, Feng AS (2005). Seasonal changes in frequency tuning and temporal processing in single neurons in the frog auditory midbrain.. J Neurobiol.

[35] Kaplan HM (1969). Anesthesia in amphibians and reptiles.. Federation Proceedings.

[36] Suckow MA, Terril LA, Grigdesby CF, March PA (1999). Evaluation of hypothermia-induced analgesia and influence of opioid antagonists in leopard frogs (*Rana pipiens*).. Pharmacol Biochem Behav.

[37] Brown LE, Brown JR (1972). Call types of the *Rana pipiens* complex in Illinois.. Science.

[38] Ehret G, Gerhardt HC (1980). Auditory masking and effects of noise on responses of the green treefrog (*Hyla cinerea*) to synthetic mating calls.. J Comp Physiol.

[39] Narins PM (1982). Effects of masking noise on evoked calling in the Puerto-Rican *Coqui* (Anura, Leptodactylidae).. J Comp Physiol.

[40] Moss CF, Simmons AM (1986). Frequency-selectivity of hearing in the green treefrog, *Hyla cinerea*.. J Comp Physiol.

[41] Ratnam R, Feng AS (1998). Detection of auditory signals by frog inferior collicular neurons in the presence of spatially separated noise.. J Neurophysiol.

[42] Jiang D, McAlpine D, Palmer AR (1997). Responses of neurons in the inferior colliculus to binaural masking level difference stimuli measured by rate-versus-level functions.. J Neurophysiol.

[43] Sakitt B (1973). Indices of discriminability.. Nature.

[44] Gai Y, Carney LH (2006). Temporal measures and neural strategies for detection of tones in noise based on responses in anteroventral cochlear nucleus.. J Neurophysiol.

[45] Goldberg JM, Brown PB (1969). Response of binaural neurons of dog superior olivary complex to dichotic tonal stimuli: some physiological mechanisms of sound localization.. J Neurophysiol.

[46] Narins PM, Wagner I (1989). Noise susceptibility and immunity of phase locking in amphibian auditory-nerve fibers.. J Acoust Soc Am.

[47] Nelson PC, Carney LH (2007). Neural rate and timing cues for detection and discrimination of amplitude-modulated tones in the awake rabbit inferior colliculus.. J Neurophysiol.

[48] Lane CC, Delgutte B (2005). Neural correlates and mechanisms of spatial release from masking: single-unit and population responses in the inferior colliculus.. J Neurophysiol.

[49] Gooler DM, Feng AS (1992). Temporal coding in the frog auditory midbrain: the influence of duration and rise-fall time on the processing of complex amplitude-modulated stimuli.. J Neurophysiol.

[50] Eggermont JJ (1989). Coding of free field intensity in the auditory midbrain of the leopard frog. I. Results for tonal stimuli.. Hear Res.

[51] Hawley ML, Melcher JR, Fullerton BC (2005). Effects of sound bandwidth on fMRI activation in human auditory brainstem nuclei.. Hear Res.

[52] Nelken I, Young ED (1994). Two separate inhibitory mechanisms shape the responses of dorsal cochlear nucleus type IV units to narrowband and wideband stimuli.. J Neurophysiol.

[53] Palmer AR, Jiang D, Marshall DH (1996). Responses of ventral cochlear nucleus onset and chopper units as a function of signal bandwidth.. J Neurophysiol.

[54] Rauschecker JP, Tian B (2004). Processing of band-passed noise in the lateral auditory belt cortex of the rhesus monkey.. J Neurophysiol.

[55] Gilbert AG, Pickles JO (1980). Responses of auditory nerve fibres in the guinea pig to noise bands of different widths.. Hear Res.

[56] Schalk TB, Sachs MB (1980). Nonlinearities in auditory-nerve fiber responses to bandlimited noise.. J Acoust Soc Am.

[57] Smalling JM, Galazyuk AV, Feng AS (2001). Stimulation rate influences frequency tuning characteristics of inferior colliculus neurons in the little brown bat, *Myotis lucifugus*.. Neuroreport.

[58] Biebel UW, Langner G (2002). Evidence for interactions across frequency channels in the inferior colliculus of awake chinchilla.. Hear Res.

[59] Escabi MA, Schreiner CE (2002). Nonlinear spectrotemporal sound analysis by neurons in the auditory midbrain.. J Neurosci.

[60] Schulze H, Langner G (1999). Auditory cortical responses to amplitude modulations with spectra above frequency receptive fields: evidence for wide spectral integration.. J Comp Physiol A.

[61] Ehret G, Schreiner CE (1997). Frequency resolution and spectral integration (critical band analysis) in single units of the cat primary auditory cortex.. J Comp Physiol A.

[62] Singh NC, Theunissen FE (2003). Modulation spectra of natural sounds and ethological theories of auditory processing.. J Acoust Soc Am.

[63] Parker AJ, Newsome WT (1998). Sense and the single neuron: probing the physiology of perception.. Annu Rev Neurosci.

[64] Schwartz JJ, Gerhardt HC (1989). Spatially mediated release from auditory masking in an anuran amphibian.. J Comp Physiol A.

[65] Bee MA (2007). Sound source segregation in grey treefrogs: spatial release from masking by the sound of a chorus.. Anim Behav.

[66] Bee MA (2008). Finding a mate at a cocktail party: Spatial release from masking improves acoustic mate recognition in grey treefrogs.. Anim Behav.

[67] Fishbach A, May BJ (2003). A neural edge-detection model for enhanced auditory sensitivity in modulated noise.. Advances in neural information processing systems.

